# Effectiveness of oral phloroglucinol as a premedication for unsedated esophagogastroduodenoscopy: A prospective, double-blinded, placebo-controlled, randomized trial

**DOI:** 10.1371/journal.pone.0255016

**Published:** 2021-08-04

**Authors:** HyeIn Jung, Hyun Jung Kim, Eun Sung Choi, Ju Yup Lee, Kyung Sik Park, Kwang Bum Cho, Yoo Jin Lee

**Affiliations:** Department of Internal Medicine, Keimyung University School of Medicine, Daegu, Republic of Korea; Hvidovre Hospital, DENMARK

## Abstract

**Background:**

Anti-spasmodic agents are commonly injected during esophagogastroduodenoscopy (EGD) to improve visualization of the gastric mucosa by inhibiting gastrointestinal (GI) peristalsis. The availability of oral anti-spasmodic agents would increase convenience. In this study, we evaluated the effectiveness of oral phloroglucinol (Flospan^®^) as a premedication for unsedated EGD.

**Methods:**

A prospective, double-blinded, placebo-controlled, randomized controlled trial was conducted in a tertiary hospital. Individuals scheduled to undergo unsedated EGD were randomly assigned to receive either oral phloroglucinol or matching placebo 15 min before EGD. The primary outcome was the rate of complete gastric peristalsis suppression. Outcomes were assessed by independent investigators according to the classification of gastric peristalsis and ease of intragastric observation at the beginning (Period A) and end (Period B) of EGD.

**Results:**

Overall, 71 phloroglucinol-treated and 71 placebo-treated participants (n = 142 total) were included. The phloroglucinol group showed significantly higher proportions of participants with complete gastric peristalsis suppression than the placebo group (22.5% vs. 9.9%, P = 0.040). The ease of intragastric observation was significantly better in the phloroglucinol group than in the placebo group at Periods A (P < 0.001) and B (P = 0.005). Patients in both groups had comparable adverse events and showed willingness to take the premedication at their next examination.

**Conclusions:**

Oral phloroglucinol significantly suppressed gastrointestinal peristalsis during unsedated EGD compared with placebo (Clinical trial registration number: NCT03342118).

## Introduction

Esophagogastroduodenoscopy (EGD), which is a basic and important modality to examine the upper gastrointestinal (GI) tract, has both diagnostic and therapeutic applications. It has also been used as an efficient tool for the surveillance of gastric cancer [1, 2]. During EGD, gastric peristalsis may significantly influence the precision of examination. Excessive peristalsis negatively affects not only the observation of simple lesions, but also other manipulations [3]. Therefore, antispasmodics have been commonly used during EGD to inhibit GI peristalsis and ultimately make endoscopic observations easier [4–6]. However, most antispasmodics, such as hyoscine butylbromide (Buscopan^®^), cimetropium bromide (Algiron^®^), atropine, and glucagon, must be injected. The administration of a drug via intravenous or intramuscular injection can cause pain and anxiety in a patient and increase medical costs [7]. Moreover, these drugs are recommended with caution as they cause potential adverse effects, such as dry mouth, urinary retention, temporary impairment of visual accommodation, palpitation, anaphylactic shock, and hyperglycemia [4, 8–11].

Phloroglucinol (1,3,5-benzenetriol; Flospan^®^) is a phenol derivative with anti-spasmodic properties [12]. It suppresses spasms by normalizing smooth muscle movement, which is excessively stimulated by acetylcholine [12, 13]. As phloroglucinol selectively inhibits smooth muscle without anti-cholinergic action, it appears to be safe as a smooth muscle relaxant in patients with glaucoma and enlarged prostate. Furthermore, oral phloroglucinol is a transparent liquid that does not interfere with the endoscopic field of view and may therefore be suitable as a pre-treatment agent. A recent study on oral phloroglucinol as a premedication for diagnostic EGD showed promising results, but that study lacked a placebo control [14]. Therefore, we aimed to evaluate the effectiveness of oral phloroglucinol as a premedication for non-sedative EGD in a randomized, double-blinded, placebo-controlled trial.

## Methods

### Trial design and patients

From September 2017 to February 2018, a prospective, double-blinded, placebo-controlled, randomized trial was carried out in a tertiary hospital. Outpatients aged 18–80 years undergoing unsedated diagnostic endoscopy were eligible for participation in the study. The following exclusion criteria were applied: (1) previous upper GI tract surgery; (2) suspected gastric outlet obstruction, deformity, or gastroparesis; (3) severe cognitive impairment; (4) hemodynamic instability; (5) suspected upper GI bleeding; (6) pregnant or lactating women; (7) upper GI mass that impaired GI motility; (8) taking medications that affected GI motility; (9) American Society of Anesthesiology (ASA) physical status classification of 4 or higher; and (10) declined to participate. These patients were considered representative of the general population undergoing non-sedative diagnostic EGD. Recruitment of participants was begun in September 2017 and ended in December 2017. At the time of the endoscopy appointment, all potential participants who were scheduled for unsedated diagnostic EGD were interviewed by an investigator in the gastroenterology outpatient clinic. An investigator explained the purpose, process and interventions of this study to the potential participants, and written informed consent was obtained from all participants before enrollment.

The study was reviewed and approved by the Institutional Review Board of Keimyung University Dongsan Medical Center (No 2017-06-043) and was registered at ClinicalTrials.gov (NCT03342118). Because the registration process took longer than expected, the study protocol was published at ClinicalTrial.gov after the enrollment commenced. The authors confirm that all on related trials for this drug/intervention are registered.

### Randomization & assignments

**Fig 1:**
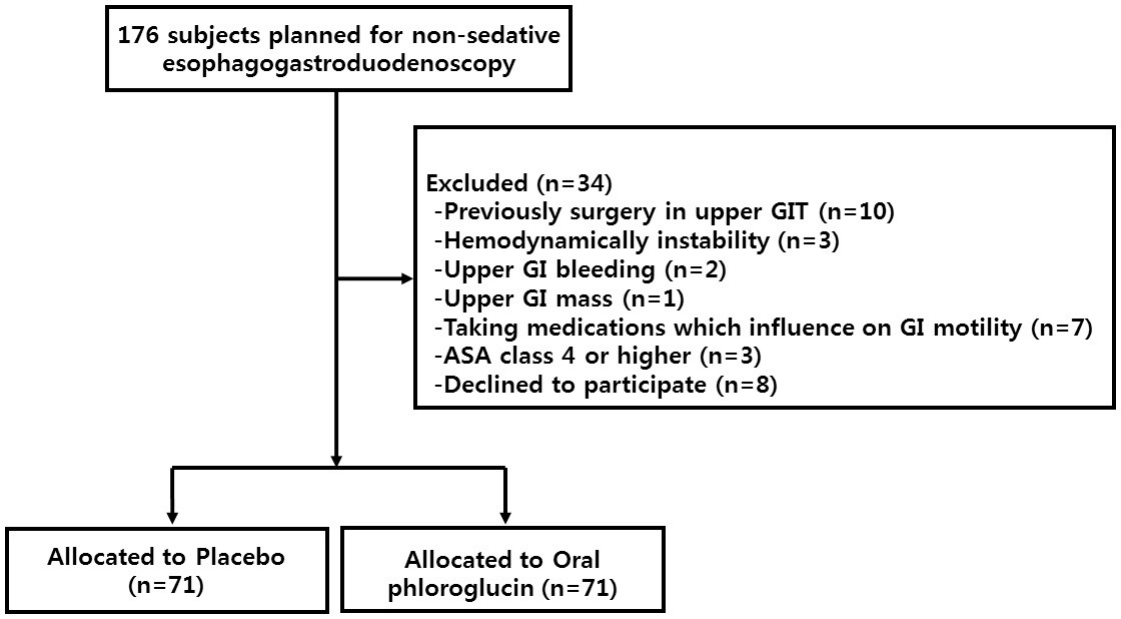
Allocation of subjects.

Flospan^®^ (Daehwa Pharmaceutical, Seoul, Korea) is a transparent liquid formulation; 20 mL of Flospan^®^ contains 160 mg of phloroglucinol. The placebo was prepared similar to the trial drug in all aspects, such as formulation, color, and flavor, but it contained only excipients. Block randomization by computer generated random number list was prepared by a statistician, who was not involved in other aspects of the study, using SAS *v*9.4 statistical software (SAS Institute, Cary, NC, USA). The sequence was masked until completion of the last participant’s enrollment. Oral phloroglucinol 160 mg (product number: 645602241 and lot number: 7001) or placebo was packaged in identical plastic pill bottles and coded according to the random sequence number. All enrolled participants were randomly assigned to either the oral phloroglucinol group or matched placebo group at a 1:1 ratio based on a randomization sequence. A flow chart of study enrollment is shown in Fig 1. Investigators responsible for participant enrollment, interviews, endoscopy performance, and video reviews were blinded to participant assignments to the study groups during the study period.

### Interventions

Before EGD, all participants were interviewed by an investigator who was blinded to participant assignments to the study groups. Details of the demographics and medical histories of patients, as well as the purpose of endoscopy were recorded. The participants were administered the assigned premedication 15 min before EGD. Immediately after the oral administration of phloroglucinol or placebo, the standard preparation process for unsedated EGD was conducted, including the application of topical lidocaine as a pharyngeal anesthetic. As the time to reach the maximum concentration (Tmax) of oral phloroglucinol is known to be 15 min after administration [15], the endoscope was inserted 15 min after the administration of oral phloroglucinol or placebo to the participants.

**Fig 2:**
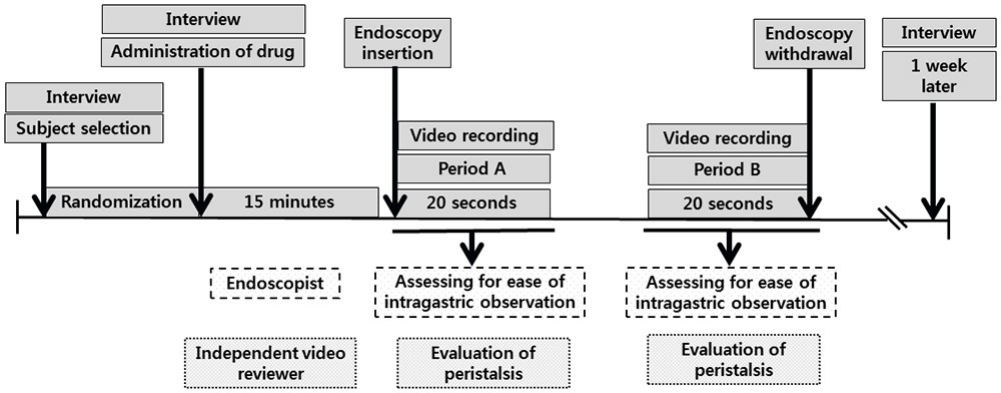
Study design.

Two expert endoscopists (HIJ and ESC) performed the endoscopy procedure. The endoscopists were blinded to participant assignments to the study groups and had no other involvement in the study. Endoscopy was performed in the morning and conscious sedation was not applied. Endoscopic videos were recorded during the EGD procedures. The videos were examined by two independent video reviewers in a masked fashion to evaluate gastric peristalsis. One week after EGD, all enrolled participants were asked about any adverse events encountered during the study period, including dry mouth, nausea, vomiting, dizziness, drowsiness, headache, dysuria, and voiding difficulty. Participant willingness to take the premedication again was also recorded with a “Yes” or “No” response. The study design is presented in Fig 2.

### Definitions and assessments

Gastric peristalsis was evaluated by dividing peristalsis into Period A and B to indicate the peristaltic effect of fluoroglucinol persisted from the beginning to the end of endoscopy. Period A was defined as the first at 20 s immediately after the insertion of the endoscope and Period B was defined the last at 20 s immediately before withdrawal of the endoscope. Gastric peristalsis was graded in two separate ways. In the first assessment, we used the “classification of gastric peristalsis,” which determined the intensity of gastric peristalsis according to the following five-point scale: Grade 1, no peristalsis; Grade 2, mild peristalsis; Grade 3, moderate peristalsis; Grade 4, vigorous peristalsis; and Grade 5, markedly vigorous peristalsis [5]. Gastric peristalsis Grades 1 and 2 were considered to be “acceptable” [5]. After EGD, two board certified endoscopists (JYL and KSP) who were familiar with the above-mentioned classification of gastric peristalsis independently assessed the intensity of gastric peristalsis by reviewing the video clips. Each time point (A and B) was graded from 1 to 5 as described with higher scores indicating more vigorous peristalsis.

The second way of assessing gastric peristalsis was the “ease of intragastric observation,” which indicated the degree to which gastric peristalsis interfered with the intragastric observation [5]. The two endoscopists who performed all of the endoscopic procedures in this trial rated peristalsis during the procedure using a score ranging from 1 to 4. The score values were defined as 1, very easy; 2, easy; 3, slightly difficult; and 4, difficult.

The criteria used to evaluate gastric peristalsis in the study are presented in Table 1. There were prepared based on existing classifications with partial modifications to enable a more objective evaluation of gastric peristalsis [5, 16]. To ensure consistency in assessing the degree of gastric peristalsis during the study period, 22 sample video clips of routine diagnostic EGD were prepared for gastric peristalsis grading as a control exercise. One week before the start of the study, the investigators responsible for video reviews (JYL and KSP) and endoscopic procedures (HIJ and ESC) independently practiced the grading system using the sample video clips.

**Table 1:** Evaluation of gastric peristalsis^a^.

**Classification of gastric peristalsis**
Grade 1: No peristalsis
No or very weak gating movement of the pyloric ring is observed, but the movement does not show a strong contraction
→ No peristalsis
Grade 2: Mild peristalsis
A circular peristaltic wave is formed in the antrum but disappears without reaching the pyloric ring, or circular contraction temporarily occurs immediately before the pyloric ring
→ Peristaltic wave does not reach the pyloric ring
Grade 3: Moderate peristalsis
A pronounced peristaltic wave is formed and reaches the pyloric ring
→ Peristaltic wave reaches the pyloric ring, which opens and closes, showing a star-like contraction as a result of the peristaltic wave
Grade 4: Vigorous peristalsis
Peristaltic wave is deep and pronounced and proceeds, strangulating the antrum
→ Peristaltic wave reaches the pyloric ring, which is totally covered by the wave, and the area exhibiting a star-like contraction protrudes towards the opening of the pyloric ring and the mucosa is pushed out from the central part of the opening
Grade 5: Markedly vigorous peristalsis
Peristaltic wave is even deeper and more pronounced, and the entire antrum appears severely strangled
→ Peristaltic wave is so deep and pronounced that the antral mucosal surface is difficult to observe because of the marked peristalsis
**Ease of intragastric observation**
Score 1: Very easy
No peristalsis is noted and no interference with observation occurs
Score 2: Easy
Mild peristalsis is noted, but observation is performed without interference
Score 3: Slightly difficult
Peristalsis is noted and slightly interferes with observation
Score 4: Difficult
Marked peristalsis is noted and makes observation difficult

^a^This classification was adapted from the criteria of HiKi et al.

### Outcomes

The primary outcome of the study was the proportion of participants with complete suppression of gastric peristalsis in both Period A and B as defined by Grade 1 classification of gastric peristalsis [5]. The secondary outcome was the proportion of participants who showed acceptable gastric peristalsis defined as Grade 1 or 2 was also compared between the two study groups. As other secondary outcomes, the ease of intragastric observation, which was rated according to the four-point scale described. We also evaluated whether the inhibition of gastric peristalsis correlated with the difficulty in intragastric observation encountered by the endoscopists. Data pertaining to adverse events after endoscopy and participant willingness to use the premedication for future procedures were collected.

### Sample size

Sample size was calculated assuming a 24.6% difference in the complete inhibition rate of gastric spasm between the placebo (11%) and trial drug (35.6%), based on a previous study [5]. With a significance level (α) of 0.05 and power of 80%, at least 121 participants were needed for this study. With a 10% expected dropout rate, we planned to enroll 134 participants to assure the detection of a significant difference in the primary outcome.

### Statistical analysis

Data were statistically analyzed using the chi-square test and independent *t*-test. When the chi-square test was performed, and the expected frequency was less than five cells in 20% or more, statistical analysis was repeated using Fisher’s exact test. The Cochran-Mantel-Haenszel test based on the Breslow-Day test was used to investigate the effect of the examined periods on gastric peristalsis. Spearman rank correlation coefficient (r_s_) was used to evaluate correlations between the grade of peristalsis and ease of intragastric observation at Periods A and B. Statistical analyses were performed using SPSS for Windows, version 21.0 (SPSS Inc., Armonk, NY, USA: IBM Corp.). Two-tailed tests were used and *P*-values less than 0.05 were considered to be statistically significant.

## Results

### Baseline characteristics

A total of 176 individuals were considered for enrollment in this study, of which 34 met the exclusion criteria and were excluded. The remaining 142 individuals were included. The participants were equally and randomly assigned into the phloroglucinol (n = 71) and placebo groups (n = 71). There was no difference between two groups (Table 2).

**Table 2:** Baseline patient characteristics (n = 142).

Characteristics	Placebo (n = 71)	Phloroglucin (n = 71)
Age, year	58.83 ± 9.68	59.37 ± 9.97
Male	39 (54.9%)	35 (49.3%)
BMI, kg/m^2^	23.69 ± 3.13	23.60 ± 3.09
Prior endoscopic procedures		
None	3 (4.2%)	2 (2.8%)
1 or 2	12 (16.9%)	16 (22.5%)
≥3	56 (78.9%)	53 (74.6%)
Endoscopic examination time, min	5.42 ± 1.28	5.16 ± 0.95
ASA score, mean ± SD	1.34 ± 0.61	1.30 ± 0.49
Comorbidity		
Diabetes mellitus	15 (21.1%)	7 (9.9%)
Hypertension	28 (39.4%)	28 (39.4%)
Cerebrovascular disease	4 (5.6%)	3 (4.2%)
Liver cirrhosis	4 (5.6%)	4 (5.6%)
Thyroid disease	0	3 (4.2%)
Kidney disease	3 (4.2%)	1 (1.4%)
Cardiovascular disease	13 (18.3%)	8 (11.3%)
Malignancy	2 (2.8%)	1 (1.4%)
Endoscopy indication		
Gastric cancer screening	58 (81.7%)	63 (88.7%)
Gastrointestinal disturbance^b^	23 (32.4%)	23 (32.4%)
History of gastric neoplasia	3 (4.2%)	5 (7.0%)
Anemia, positive result of stool occult blood	1 (1.4%)	1 (1.4%)
Personal history of malignancy	3 (4.2%)	0
Body weight loss	0	1 (1.4%)
Biopsy	41 (57.7%)	44 (62.0%)

Data are n (%) or mean ± SD,

BMI, Body mass index; ASA, American Society of Anesthesiologists comorbidity

^b^Gastrointestinal disturbance includes epigastric soreness, pain, discomfort, acid reflux, dyspepsia, and nausea, etc).

### Degree of gastric peristalsis

**Fig 3:**
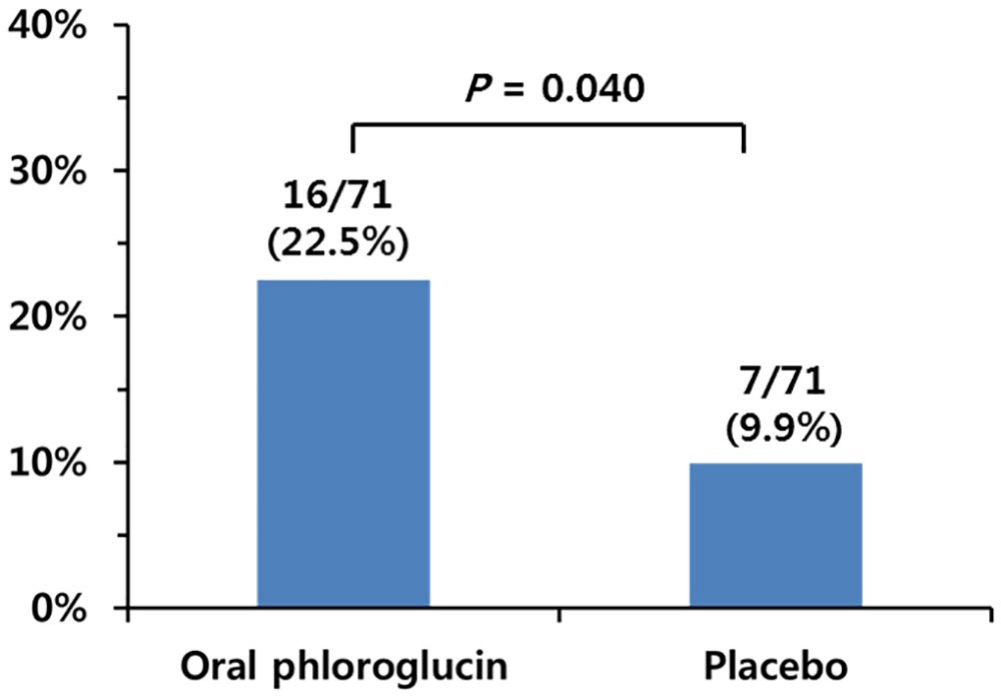
The proportions of subjects with complete suppression (Grade 1) of gastric peristalsis.

**Fig 4:**
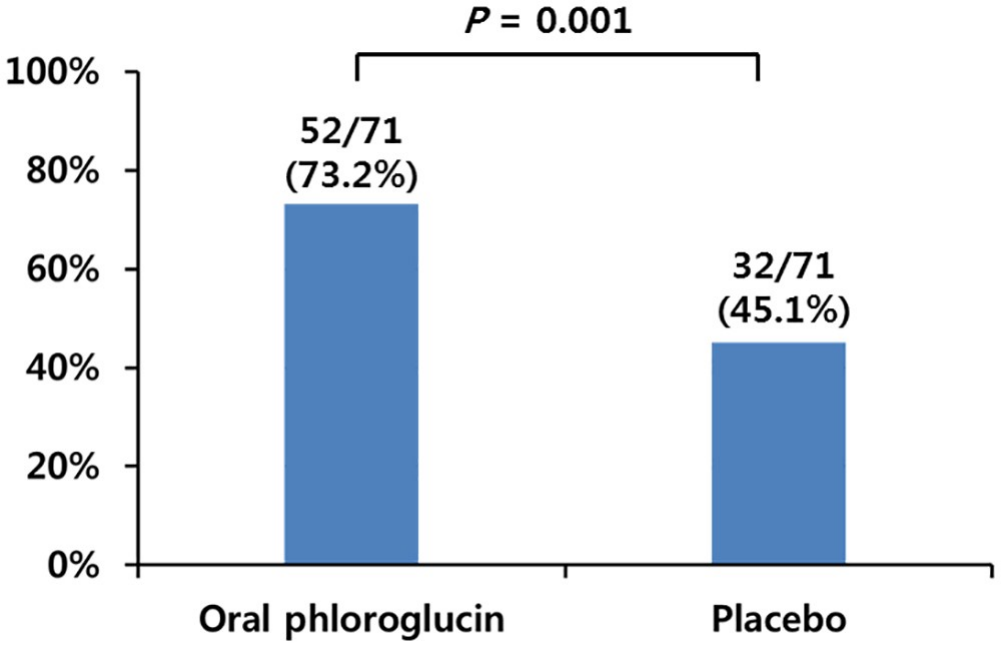
The proportions of subjects who showed acceptable peristalsis^d^ in Phloroglucin and Placebo group. ^d^Acceptable peristalsis includes grade 1 and grade 2.

As shown in Fig 3, the phloroglucinol group had a significantly higher percentage of participants who showed a complete suppression of gastric peristalsis (Grade 1) than the placebo group (22.5% vs. 9.9%, P = 0.040). As shown in Fig 4, the percentage of participants with acceptable peristalsis, defined as Grades 1 and 2, was also significantly higher in the phloroglucinol group than in the placebo group (73.2% vs. 45.1%, P = 0.001). The mean score for gastric peristalsis was significantly lower in the phloroglucinol group than in the placebo group for Periods A (1.90 ± 0.91 vs. 2.48 ± 1.13, *P* = 0.001) and B (2.09 ± 1.13 vs. 2.54 ± 1.11, *P* = 0.018) (Table 3).

**Table 3:** Peristaltic grade^e^ and the ease of intragastric observation^f^.

	Phloroglucin (n = 71)	Placebo (n = 71)	Mean difference	
			*vs* Placebo (95% CI)	*P value*^*g*^
Peristaltic grade				
Period A, mean ± SD	1.90 ± 0.91	2.48 ± 1.13	-0.58 (-0.92 to -0.24)	0.001
Period B, mean ± SD	2.09 ± 1.13	2.54 ± 1.11	-0.45(-0.82 to -0.08)	0.018
Ease of intragastric observation‡				
Period A, mean ± SD	1.87 ± 0.77	2.52 ± 0.77	-0.65 (-0.90 to -0.39)	<0.001
Period B, mean ± SD	2.03 ± 0.94	2.44 ± 0.75	-0.41 (-0.69 to -0.13)	0.005

^e^Score range from 1 to 5, and higher scores indicates more vigorous peristalsis.

^f^Scores range from 1 to 4, and a higher score indicates that intragastric observation was further inhibited by peristalsis.

^*g*^*P values* were calculated by independent *t*-test. In Cochran-Mantel-Haenszel test, *P value* based on the Breslow-Day test was not statistically significant.

### Subjective endoscopist discomfort level for intragastric observation

A comparison of the ease of intragastric observation between the two study groups is shown in Table 3. The mean score for the ease of intragastric observation was significantly lower in the phloroglucinol group than in the placebo group for both Periods A (1.87 ± 0.77 vs. 2.52 ± 0.77, *P* < 0.001) and B (2.03 ± 0.94 vs. 2.44 ± 0.75, *P* = 0.005).

To determine whether the peristalsis grade scale used in the trial correlated with the subjective endoscopist discomfort level for intragastric observation in real-world clinical practice, a four-grade scale of intragastric observation was compared with the classification of gastric peristalsis determined by the video reviewers. Table 4 summarizes the correlation analysis between the degree of gastric peristalsis and ease of intragastric observation. The degree of gastric peristalsis positively correlated with the ease of intragastric observation, which was assessed by the independent endoscopists. Specifically, the r_s_ value was 0.706 at Period A, which indicated a high positive correlation (*P* < 0.001) and was 0.643 at Period B, suggesting a moderately positive correlation (*P* < 0.001) [17].

**Table 4:** Correlation between the classification of peristalsis and ease of intragastric observation.

Time period	Classification of peristalsis	Ease of intragastric observation, n (%)				Spearman rank Value (r_s_)	*P value*^h^
		Very easy	Easy	Slightly difficult	Difficult		
Period A	No peristalsis (n = 40)	21 (52.5)	15 (37.5)	3 (7.5)	1 (2.5)	0.706	<0.001
	Mild peristalsis (n = 59)	6 (10.2)	49 (83.1)	4 (6.8)	0		
	Moderate peristalsis (n = 24)	0	5 (20.8)	17 (70.8)	2 (8.3)		
	Vigorous peristalsis (n = 14)	0	2 (14.3)	7 (50.0)	5 (35.7)		
	Markedly vigorous peristalsis (n = 5)	0	0	2 (40)	3 (60)		
Period B	No peristalsis (n = 33)	21 (61.8)	8 (23.5)	5 (14.7)	0	0.643	<0.001
	Mild peristalsis (n = 64)	7 (11.1)	44 (69.8)	11 (17.5)	1 (1.6)		
	Moderate peristalsis (n = 21)	2 (9.5)	5 (23.8)	12 (57.1)	2 (9.5)		
	Vigorous peristalsis (n = 15)	0	3 (20.0)	11 (73.3)	1 (6.7)		
	Markedly vigorous peristalsis (n = 5)	0	0	2 (22.2)	7 (77.8)		

Data are presented as n (%).

^h^P values were calculated by Spearman rank correlation.

### Safety and future willingness of participants to take the assigned premedication

Table 5 shows adverse events and participants’ willingness to re-intake the assigned premedication for future procedures. There was no statistically significant difference in the rate of adverse effect occurrence between the phloroglucinol and placebo groups. The most common adverse effect after the administration of oral phloroglucinol was dry mouth, which was experienced by six of the participants (8.5%) who received oral phloroglucinol compared with five participants (7.0%) in the placebo group. The dry mouth symptom improved within 1 h after the completion of EGD in all participants. Three participants in the phloroglucinol group (4.2%) and four participants in the placebo group experienced dizziness; however, the symptoms were mild and self-limited within several hours. All adverse effects were considered non-serious, and all cases lasted less than a day. In addition, most participants reported a willingness to retake the medication for any future EGD, which did not significantly differ between the two study groups.

**Table 5:** Comparisons of adverse events and the willingness of subjects to re-intake the assigned premedication.

Characteristics	Placebo (n = 71)	Phloroglucin (n = 71)	*P value*^i^
Dry mouth	5 (7.0%)	6 (8.5%)	1.000
Headache	1 (1.4%)	2 (2.8%)	0.560
Dizziness	4 (5.6%)	3 (4.2%)	0.698
Abdominal pain	2 (2.8%)	2 (2.8%)	1.000
Nausea, vomiting	3 (4.2%)	2 (2.8%)	0.649
Voiding difficulty	1 (1.4%)	1 (1.4%)	1.000
Blurred vision	0	0	1.000
Willingness to re-intake	67 (94.4%)	70 (98.6%)	0.172

Data are presented as n (%).

^i^*P* values were calculated by chi-squared test or Fisher’s exact tests.

## Discussion

In this present study, the effectiveness of oral phloroglucinol was evaluated and compared with that of a placebo during EGD in individuals scheduled to undergo unsedated EGD. Oral phloroglucinol was significantly more effective than the placebo in suppressing gastric peristalsis during EGD, and it made the procedure less difficult for the endoscopists to perform. Our results also revealed that the anti-peristaltic effect of oral phloroglucinol positively correlated with the subjective discomfort level of the endoscopist with the intragastric evaluation.

Recent advances in endoscopic technology have enabled earlier diagnoses with a relatively low level of invasiveness, which has ultimately improved patient survival. As the GI tract is essentially a peristaltic organ, GI motility can be exaggerated during endoscopic examinations. Vigorous peristalsis of the GI tract may interfere with detailed observations of the mucosal surface and prolong procedure time due to the need to wait for the peristaltic wave to subside. Furthermore, in patients who undergo unsedated EGD, peristalsis may lead to patient discomfort or most importantly may hinder endoscopic yield, resulting in a missed biopsy. In this regard, several agents have been investigated in an effort to improve gastric peristalsis during EGD; however, these investigations have generated conflicting results and insufficient evidence supporting the clinical usefulness of the agents [8–10, 18]. Phloroglucinol is an organic compound that has nonspecific antispasmodic properties [15]. Although the pharmacodynamics of phloroglucinol are not yet clearly elucidated, it is considered to directly relax smooth muscle by inhibiting voltage-dependent calcium channels [19]. Based on its spasmolytic activity, phloroglucinol has been investigated in various diseases to treat spasmodic pain and irritable bowel syndrome (IBS) [20–22]. Despite the widespread prescription of phloroglucinol, especially in Europe, data to support its clinical utility are limited [23].

Before the study, the efficacy of oral phloroglucinol as a premedication for EGD had not been extensively evaluated. Only one study compared the suppression of peristalsis during diagnostic EGD between oral phloroglucinol and intravenous cimetropium bromide [14]. Oral phloroglucinol was inferior to cimetropium bromide in inhibiting gastric peristalsis, but the difference was not clinically significant. Therefore, the authors postulated that oral phloroglucinol can be used as an antispasmodic agent during EGD with similar antispasmodic efficacy as cimetropium bromide [14]. Consistent with this previous report, our study showed that oral phloroglucinol significantly suppressed gastric peristalsis in patients undergoing unsedated EGD and that it demonstrated acceptable safety. By using oral phloroglucinol as a premedication for EGD, peristalsis was completely inhibited (Grade 1) in in 22.5% of the study participants and clinically acceptable peristalsis (Grades 1 and 2) was observed in 73.2% of the participants.

In terms of safety, phloroglucinol is believed to be free from anti-cholinergic adverse effects that would limit its routine clinical utility [21]. In this current study, oral phloroglucinol exhibited a good safety profile as there was no statistically significant difference in adverse events between oral phloroglucinol and the placebo. Most adverse events were mild and spontaneously resolved with no additional management requirement. These findings are consistent with a previous study that demonstrated no serious adverse events during the study period [14]. Furthermore, the incidence of dry mouth was significantly lower in patients treated with phloroglucinol than in those treated with cimetropium bromide during diagnostic EGD [14]. These safety outcomes are noteworthy as other antispasmodics have been previously reported to cause various adverse events. For instance, anticholinergics, such as atropine and hyoscine-N-butylbromide, can result in decreased saliva secretion, blurred vision, glaucoma, palpitations, and allergic reactions [24], and glucagon has been reported to be associated hyperglycemia, reactive hypoglycemia, and allergic reactions [8, 25, 26].

Numerous studies have reported an increased risk of sedation-related adverse events in elderly patients when compared with younger patients, such as hypoxia, hypotension, and aspiration [27–30]. Therefore, unsedated EGD may be considered the best option for elderly individuals; however, adverse events caused by conventional antispasmodics are particularly problematic in elderly patients who have a high prevalence of comorbidities. Although we did not specifically target the elderly population in this study, oral phloroglucinol showed good safety in these patients. Considering its safety and convenience of administration, oral phloroglucinol may be one of the best options for patients undergoing unsedated EGD, especially elderly patients.

Our study had some limitations. First, only individuals undergoing unsedated EGD were included. As intravenous antiplasmodics can be easily administered using existing intravenous routes available for sedative agents, our data may not fully validate the utility of oral phloroglucinol as a premedication for EGD. Further studies will be needed to establish the effectiveness of oral phloroglucinol in a wider variety of endoscopic procedures, including EGD with sedation. Second, we did not evaluate the satisfaction of patients with oral phloroglucinol. Since it is well known that oral formulations are expected to reduce patient discomfort and anxiety associated with needle injections [7], additional research should be performed regarding patient satisfaction with oral phloroglucinol in line with current medical trends of patient-centered medicine.

Despite these limitations, our study has its strengths. First, to the best of our knowledge, this is the first prospective, double-blinded, randomized controlled trial to compare the efficacy of oral phloroglucinol in suppressing gastric peristalsis with that of a placebo. Second, a recently reported modified endoscopic classification system [5] was adopted in our study to grade gastric peristalsis. We acknowledge that the lack of validation of this modified classification system can also be considered as a drawback. However, this classification allowed a more objective and convenient evaluation of gastric peristalsis. In addition, peristalsis was graded by two independent investigators, thereby enhancing the objectivity of the study and reducing potential bias. Furthermore, we strictly controlled the blinding of participant assignments to the study groups and peristalsis was graded by reviewing recorded endoscopic video clips. To ensure objective evaluation, the investigators practiced the grading system by watching sample video clips before the start of the actual study.

In conclusion, our findings confirmed that the administration of oral phloroglucinol significantly suppressed gastric peristalsis and improved the ease of observation by the endoscopists during unsedated EGD. Further studies are needed to validate the efficacy of this medication in a variety of endoscopic therapeutic procedures in real world clinical practice. However, considering its efficacy, good safety profile, and convenience of administration, the routine use of oral phloroglucinol should be considered for patients undergoing unsedated EGD.

## Supporting information

S1 Data (XLSX)

S1 File (DOCX)

S2 File (DOCX)

S3 File (DOC)
