# Supplementary material for: Effectiveness of oral phloroglucinol as a premedication for unsedated esophagogastroduodenoscopy: A prospective, double-blinded, placebo-controlled, randomized trial
Source: PLoS One. 2021 Aug 4;16(8):e0255016. doi: 10.1371/journal.pone.0255016 (PMC8336830; doi:10.1371/journal.pone.0255016)
Supplement: S1 File — (DOCX) [file pone.0255016.s002.docx]

**연구계획서**

**1. 연구과제명**

비수면 진단 위내시경 검사의 전처치제로서의 Phloroglucin (후로스판®)의 효과

: 단일기관, 전향적, 양측눈가림, 무작위 배정 임상연구

**2. 연구기관명**

계명대학교 동산의료원, 대구광역시 달서구 달구벌대로 1035

**3. 연구책임자, 공동연구자, 연구 담당자의성명및직명**

임상연구책임자 – 이유진, 조교수, 계명대학교 동산의료원소화기내과

공동연구자 – 정혜인, 임상교수, 계명대학교 동산의료원 소화기내과

김현중, 임상교수, 계명대학교 동산의료원 소화기내과

최은성, 임상교수, 계명대학교 동산의료원 소화기내과

이주엽, 조교수, 계명대학교 동산의료원 소화기내과

박경식, 교수, 계명대학교 동산의료원 소화기내과

조광범, 교수, 계명대학교 동산의료원 소화기내과

**4. 연구간호사 및 임상약 관리약사/의료기기 관리자의 성명 및 직명**

계명대학교 동산의료원 소화기내과 연구간호사 김채영, 053-250-8034

**5. 지원(의뢰)기관명, 주소, 담당자명 및 연락처**

**:** 해당없음.

**6. 연구 목적**

**6.1. 1차 연구 목적 (Priamry end point)**

비수면 진단위내시경 검사시 전처치제로 Phloroglucin (후로스판®)을 투여하였을 때 위약 (Placebo)를 투여한 군에 비해 위 연동운동의 정도에 차이가 발생하는지 조사한다.

**6.2. 2차 연구목적 (Secondary end point)**

1) Phloroglucin (후로스판®)을 투여한 군과 위약 (placebo)을 투여한 군에서 약제 투여후 위 연동운동으로 인한 검사자의 불편감에 차이가 발생하는지 조사한다.

2) Phloroglucin (후로스판®)와 관련된 부작용 유무를 조사하고자 한다.

3) Phloroglucin (후로스판®)을 투여한 군과 위약 (placebo)을 투여한 군에서 다음 검사시 같은 내시경 전처치제를 재복용할 의사의 차이가 발생하는지 조사한다.

**7. 대상질환**

비수면 진단 위내시경을 시행하는 환자

**8. 연구예정기간**

IRB 승인일로부터 2018.06.30까지

**9. 임상시험 대상자의 선정/제외 기준**

본 임상시험은 선정기준에 모두 합당하고 제외기준에 하나도 해당되지 않는 환자를 대상으로 한다.

**9.1. 선정기준**

1) 만 18세 이상 80세 이하 남녀

2) 진단 또는 검진 목적으로 비수면 위내시경 검사를 시행하는 자

3) 임상시험의 지시사항 및 설문지를 이해하고 따를 수 있으며, 의사소통에 문제가 없는 자

4) 본 임상시험에 자의로 참여를 결정하고 서면 동의한 자

**9.2. 제외기준**

1) 상부위장관의 수술을 시행한자

2) 심한 위 출구 폐쇄 혹은 기형 도는 위마비증이 있는 경우

3) 심한 인지능력의 장애가 있는 경우

4) 혈류역학적으로 불안정한 경우

5) 상부위장관 출혈로 내시경적 지혈이 필요한 경우

6) 임신 혹은 수유중인 여성

7) 상부 위장관 운동장애를 유발하는 종양이 있는 경우

8) 위장관 운동에 영향을 주는 약제 복용중인 경우

9) ASA (American Society of Anesthesiology)classification class 4 이상인 경우

10) 환자가 거부하는 경우

**9.3. 시험중지 및 탈락기준**

1) 환자가 동의를 철회한 경우

2) 환자가 내시경 검사를 임의 중단하거나 설문지를 작성하지 않는 경우

**9.4. 선별절차**

시험 책임자 또는 시험 담당자는 어떠한 임상시험계획서에 명시된 절차를 시행하기 전에 모든 환자들로부터 본 시험에 대한 서면 피험자 동의서를 받는다. 등록을 위해 피험자는 선정/제외 기준을 충족하여야 한다.

**10. 연구대상수 (본원/전체) 및 산출 근거**

연구대상수는 기존의 연구결과 (Gastrointest Endosc 2011;73:932-41)에 의한 시험군에서 위연동운동이 완전히 제어된 환자의 비율이 35.6%, 대조군에서 위연동운동이 완전히 제어될 환자의 비율을 11%로 알려져 있어 두군의 차이가 24.6%라는 가정하에 계산되었다. 검정력80%, 유의수준 0.05로 최소 121명의 참가자가 이 연구에 필요하며 drop rate 10%를 기준으로 sample size 계산공식을 이용하여 sample size 총 134명을 등록할 계획하에 연구를 진행한다.

**11. 연구 대상의 시험참가 동의**

반드시 연구 개시 전에 환자 및 필요한 경우 보호자에게 본 연구에 대한 충분한 설명과 이해 후 서면으로 받도록 한다.

**12. 연구 대상의 연구 적용 방법**

비수면 진단 위내시경을 시행하는 외래 또는 입원환자 중 선정 기준에 부합하는 사람들 가운데 연구에 동의하는 경우 본 연구에 참여시킨다.

**12-1. 취약한 피험자 등록**

1) 취약한 시험대상자 포함 사유

비수면 위내시경 검사는 수면 위내시경 검사보다 안전한 검사이며, 최근 3차병원에서 비수면 위내시경을 시행하는 환자가 많지 않아, 원활한 시험대상자 등록을 위한 방안의 일환으로 취약한 시험대상자를 대상자 범위에 포함시키고자 한다. 또한, 본 임상시험은 '서면 동의 취득일 기준 연령이 만18세 이상 80세이하가 참여할 수 있으므로, 만 70~80세인 시험대상자가 IRB에서 제시한 취약한 환경의 시험대상자 중 노인(70세 이상)을 포함시키고자 한다. 이에 대한 대책은 아래와 같다.

2) 취약한 시험대상자에 대한 대책

① 취약한 시험대상자가 본 임상시험에 참여를 원하는 경우, 시험자는 해당 시험대상자가 취약한 환경에 속해 있음을 충분히 설명할 것이며, 동의를 얻기 전에 시험대상자 또는 대리인이 임상시험의 세부사항에 대해 질문하고 참여 여부를 결정할 수 있도록 충분한 시간과 기회를 줄 것이다.

② 모든 시험대상자는 자발적으로 임상시험 참여 여부를 결정할 것이며, 시험자는 어떠한 경우에도 시험대상자에게 임상시험 참여 또는 참여 지속을 강요하거나, 부당하게 영향을 끼치지 않을 것이다.

③ 모든 자료는 요약 또는 모든 개인식별정보를 제거함으로써 시험대상자의 신원이 보호되도록 할 예정이다.

④ 취약한 연구대상자가 본 시험에 참여하도록 유도하기 위해 개별적으로 접촉하거나 부가적인 인센티브를 제공하지 않겠다.

**13. 연구의 안전성 및 유효성**

현재 임상에서 보편적으로 사용 중인 약제이므로 본 임상 시험으로 인해 추가되는 안전성 및 유효성 문제는 없으나, 약제 복용으로 인한 구역, 구토증상이 있을 수 있다. 책임 연구자는 검사 이후 환자의 불편감 및 부작용 여부를 확인할 것이다.

**14. 시험약 관리자 및 약물 투여 계획**

Phloroglucin (후로스판®)을 제조하는 대화제약㈜ 회사에서 제작한 모양이 색상과 성상이 동일한 위약을 이용하여 이중 맹검으로 진행한다. 제조한 약제는 임상 책임연구자가 관리한다**.**

**15. 임상시험방법**

**15.1 임상시험의 설계**

1) 본 임상시험은 3차 종합병원 단일기관, 전향적, 양측눈가림, 무작위배정 임상시험으로 진행하고자 한다.

2) 자의에 의해 임상시험 동의서에 서명한 18세에서 80세 연령의 진단적 비수면 내시경을 시행받는 환자가 시험대상자이다.

3) 선정/제외기준에 적합한 대상자에 한하여 시험군 또는 대조군에 1:1로 무작위 배정되어 시험약 (Phloroglucin 액 160mg)/대조약 20ml을 위내시경 전처치로써, 위내시경 검사 15분전에 경구 복용한다. 복용 직후 인두 마취목적의 리도카인 점적과 같은 기존 비수면 위내시경의 준비과정이 수행된다.

4) 내시경 삽입은 약제 복용 15분 후에 이루어지며 두명의 내시경 전문의가 내시경을 수행하며 내시경 전문의들은 참가자들의 배정에 대하여 알지 못하며 어떠한 다른 개입도 하지 않는다.

5) 내시경 영상이 시술 도중 녹화되며 영상은 위연동운동을 평가하기 위하여 두명의 독립적인 영상 판독가들에 의해서 평가된다.

6) 내시경 시행 일주일 뒤 모든 참가자들은 연구기간 동안 구갈, 오심, 구토, 어지러움, 기면, 두통, 배뇨곤란, 배뇨통과 같은 다른 부작용이 발생하였는지 설문을 받게 된다. 그리고 참기자들은 전처치제를 다음에도 재복용할 의사가 있는지에 대해서 네 혹은 아니오로 설문을 작성한다.

**15.2. 임상시험용 의약품 투여량 및 투여방법**

시험약 혹은 대조약을 160mg을 내시경 검사 15분전에 경구 투약한다.

*투약 근거: Phloroglucin 액은 성인 1회 투여 용량이 160mg이며, 복용후 10-15분 후 효과가 나타나며, Tmax는 15분이다.

**15.3 평가항목**

1) 위 연동운동과 검사자의 불편감의 평가

1. 위연동운동은 내시경 삽입 직후 20초(Period A) 와 내시경 회수 직전 20초(Period B) 두 시점에서 평가되어진다.
2. 내시경 연동운동은 두가지 독립된 방법으로 점수화한다,

- 첫번째는 다음과 같은 5단계에 따라 위 연동운동의 강도를 결정하는 위연동운동 분류법을 사용한다.

: Grade 1, 연동운동 없음; Grade 2, 약한 연동운동; Grade 3, 중등도 연동운동; Grade 4, 중증 연동운동; Grade 5, 아주 심한 중증의 연동운동.

내시경 검사 후 연동운동의 분류법에 숙련된 두명의 내시경 전문의(JYL, KSP)가 독립적으로 비디오영상을 관찰함으로써 위연동운동의 강도를 평가한다.

각 시점에서 좀더 격렬한 연동파를 나타내는 시점에 1에서 5점 사이의 점수를 측정한다.

- 두번째 위 연동운동을 평가하는 방법은 위내 관찰의 용이성으로 위 연동운동이 위내부 관찰을 방해하는 정도를 나타낸다.

내시경을 수행하는 두명의 내시경 전문의가 1에서 4점 사이의 점수로 기록하게 되며 점수는 1점 매우 쉬움, 2점 쉬움, 3점 약간 어려움, 4점 어려움으로 매겨진다.

1. 위연동운동을 평가하는 기준은 표1에 제시되어있다. 위 연동 운동의 더 객관적인 평가를 가능하게하기 위해 기존 분류를 기초하여 부분수정된 분류법을 제시하였다.
2. 위 연동운동의 강도를 평가할 때 일관성 유지를 위하여 진단 위내시경 22개의 샘플 비디오 클립을 준비 하였다. 연구 시작 1 주일 전에 비디오 검토 (JYL 및 KSP) 및 내시경 수행(HIJ 및 ESC)를 담당하는 시험자는 샘플 비디오 클립을 사용하여 등급 시스템을 독립적으로 수행하였다.

**Table 1. 위 연동운동의 평가***

| **위연동운동 분류법** |
| --- |
| ***Grade 1: No peristalsis*** |
| Pyloric ring의 움직임이 없거나 거의 관찰되지 않으며 강한 수축이 없음 |
| ***Grade 2: Mild peristalsis*** |
| 원형의 연동파가 antrum에 관찰되나 pyloric ring까지 도달하지 않는 경우, 혹은 Pyloric ring에 도달하기 직전 일시적으로 원형의 수축을 보임 |
| ***Grade 3: Moderate peristalsis*** |
| 확연한 연동파가 pyloric ring까지 도달하며 수축시 별모양으로 관찰됨 |
| ***Grade 4: Vigorous peristalsis*** |
| 깊고 확연한 연동파가 antrum의 strangulation양상으로 관찰되며 별모양 수축을 보이는 부위가 pyloric ring 입구쪽으로 돌출되고 입구 중앙으로부터 점막이 밀려져 나옴 |
| ***Grade 5: Markedly vigorous peristalsis*** |
| 깊고 확연한 연동파가 심하게 있으며 전체 antrum이 뒤틀려 보여 점막 표면을 관찰하기 힘듦 |
| **내시경 검사시 위 내부 관찰의 편의성** |
| ***Score 1: Very easy*** |
| → 연동운동이 관찰되지 않고 검사시 불편감 없음 |
| ***Score 2: Easy*** |
| → 연동운동이 다소 있으나 검사시 불편감은 없음 |
| ***Score 3: Slightly difficult*** |
| → 연동운동이 있으며 검사시 다소 불편감 있음 |
| **Score 4: Difficult** |
| → 검사에 방해될 정도의 확연한 연동운동이 있음 |

*위 분류법은 HiKi 등이 제시한 기준을 채택하였다,

**15.4. 대상자 설문지**

내시경 검사시행 전과 후에 대상자는 설문지를 평가한다. 평가 내용은 아래와 같은 내용을 포함하고 있다.

1) 대상자 기본정보-성별,나이, 키,몸무게, 복부 수술력및 동반 질환

2) 위내시경 검사 시행이유, 과거 위내시경 검사 시행 횟수

3) 시험약 혹은 대조약 복용후 부작용 발생 유무

4) 다음 검사시 같은 내시경 전처치제를 재복용할 의사 유무

**16. 무작위 배정 방법**

**16.1 무작위 배정표의 작성**

무작위 배정은 SAS system의 randomization program으로 발생된 난수 (A,B의 random number)의 순열을 임상시험 대상자 번호 1번부터 순차적으로 적용시킨 표이다 (예; A군=시험군, B군=대조군). Block size는 본 계획서에서는 명시하지 않기로 한다.

**16.2. 무작위 배정 방법**

임상시험용 의약품 제조자는 임상시험용 의약품의 포장 및 라벨링 시에 무작위 배정표에 따라 시험약, 대조약을 적절하게 배정, 포장하고 해당 배정번호 (임상약 번호)를 라벨링하여야 한다.

시험자는 선정/제외기준에 적합한 임상시험 대상자에 의해 무작위배정 되는 순서대로 배정번호를 부여하고, 다른 연구자가 시험자가 배정한 배정번호와 일치하는 배정번호가 라벨된 약제를 임상시험 대상자에게 공급함으로써 무작위 배정이 이루어진다. 무작위 배정은 시험군과 대조군 비율이 1:1로 배정될 수 있도록 한다.

**16.3. 양측 눈가림 방법 및 유지**

본 임상시험에서는 다음과 같이 양측 눈가림을 실시한다. 시험약과 대조약의 성상과 복용방법이 동일하므로 대상자와 임상시험용 의약품의 불출 및 반납, 순응도 확인 등을 담당하는 시험자는 눈가림이 진행된다. 또한 유효성을 평가하는 독립된 평가자와 내시경의사는 눈가림이 진행된다. 본 연구의 내시경의사는 임상약 불출, 반납 등 임상시험용 의약품과 관련된 모든 업무를 수행할 수 없으며, 이를 담당하는 시험자 또는 대상자와 논의할 수 없다.

**17. 모니터링 계획**

연구책임자와 담당자간의 회의를 통해 자료 안전모니터링을 시행하며 수집된 자료의 보관기관은 연구종료 후 3년이다.

**18. 연구 수행 일정**

본 연구는 시험대상자 선정 및 연구 진행 및 종료가 모두 내시경 검사 당일 이루어지며 총 연구기간은 IRB 승인일로부터 12개월 간이다. 처음 3개월간 기관별 연구윤리심의위원회 심사를 종료하고 이후 6개월간 연구를 진행한다. 이후 1개월간 자료 취합 및 데이터 통계분석을 시행하고 최종 2개월에는 논문을 작성한다.

| 月 | 6 | 7 | 8 | 9 | 10 | 11 | 12 | 1 | 2 | 3 | 4 | 5 |
| --- | --- | --- | --- | --- | --- | --- | --- | --- | --- | --- | --- | --- |
| 1. IRB심의  2. 환자등록시작  3. 연구진행  4. 환자등록종료  5. 결과분석  6. 논문작성 | O | O | O | O  O | O  O | O  O | O  O | O  O | O  O | O  O | O | O |

**19. 임상시험 중지기준**

1)연구 조기 종료: 환자의 등록이 너무 빨라서 목표한 피험자의 수에 도달하여 충분히 본 연구를 시행할 수 있는 경우

2) 연구 종료: 계획대로 연구가 진행되어 종료 시점에 도달한 경우

**20. 부작용을 포함한 안전성의 평가기준, 평가방법 및 보고방법**

**20.1. 검사전후 임상적 안정성을 확인한다.**

환자에게 복용하도록 하는 약제는 현재까지 유의한 부작용이 보고된 바 없다. 연구 방법상 부작용 및 위해가 예상되지 않으나 경미한 위장관 증상 유무에 대해 추적 진료 시 확인할 것이다.

**20.2. 피험자의 안전보호에 관한 대책**

임상시험 실시기관은 본 시험계획서에 규정된 대로 임상시험이 적절히 진행될 수 있도록 임상시험에 필요한 설비와 전문인력을 갖추고 피험자의 안전보호에 만전을 기해야 한다. 시험담당자는 본 계획서에 명시된 이상반응 및 주의사항 등에 대하여 사전에 충분히 숙지하고 시험도중 중대한 이상반응 등이 발생할 경우에는 필요에 따라 해당피험자의 임상시험을 중지하고 적절한 조치를 취한 후 임상연구심의위원회에게 통보하여야 한다.

**21. 피해자 보상에 대한 규약**

본 연구 실시 중에 연구로 인한 이상반응 등 예기치 않은 사고가 발생하여 치료 또는 입원이 요구되는 경우 그 인과관계가 인정되면 연구자가 그 비용을 부담한다. 단, 발생한 이상반응에 대하여는 피험자가 연구자에게 즉각적인 연락을 취하여 상태가 악화되지 않도록 하여야 한다.

**22. 연구의 목적을 위해 적용되는 의약품 또는 기타 시술의 지속적인 안전성 모니터링 계획**

**22.1.** **환자의신분(Identification)**

임상시험에 선발된 모든 환자의 성명머리 글자와 생년월일을 시간순으로 첫방문시 환자일지(patient log)에 기록한다. 환자가 임상시험 참가에서 제외될경우, 그 이유를 환자일지 (patient log)에 기록한다. 독립된 공간에서 상기 연구에 대해 환자에게 면담, 설명 및 동의를 취득한다. 각 환자는 등록시 배정번호를 배정받게되며, 이 배정번호와 성명 머리글자가 환자증례 기록지에 기입된다.

**22.2. 자료의기록(Recording of Data)및 보관**

모든 해당자료는 증례기록지에 입력되며 접근이 제한된 컴퓨터에 저장하고 잠금 장치에 보관한다. 모든 해당 자료는 시험이 종료된 후 최소 5년 동안 보관한다.

**22.3. 자료안전 모니터링 계획**

현재 임상에서 진단 및 치료를 위해 광범위하게 이용되고 있는 조직 검사 및 약제복용 외에 추가적으로 환자에게 이루어지는 시술이나 약물 주입은 없으므로 예상 되는 이상반응이나 특이 주의사항은 없다고 판단된다. 따라서 모니터링을 위한 안전 점검자는 연구자로 가능할 것이다. 동의서에 연구에 참여하신 후라도 언제든지 취소를 할 수 있음이 명시되어 있고, 연구 참여 동의에 대해서는 외래 진료 방문 시마다 지속적으로 확인한다.

**23. 증례 기록서의 작성 및 대상자의 비밀보장**

본 연구 중에 수집된 모든 자료는 별첨의 증례기록서(CRF)에 연구자의 책임하에 기록되어야 한다. 본 연구는 모든 대상자의 비밀을 보장하기 위하여 연구과정에서 부여한 대상자 식별코드번호에 의해 기록 및 평가를 실시하고 논문발표 등 결과의 외부 발표시에도 모든 대상자의 비밀은 보장되어야 한다.

**24. 통계 처리 방법**

1) 임상 증상의 호전에 대한 분석은 chi square test 및 독립 T-test를 이용한다.

2) 카이제곱 검정 시행 시 기대빈도가 5 미만인 cell 이 20%를 넘을 경우에는 카이제곱 검정을 대신하여 피셔의 정확한 검정으로 통계 분석을 시행한다.

3) 스피어 맨 순위 상관 계수 (r)를 사용하여 Period A 및 B에서 연동운동 강도와 위내 관찰의 용이성 사이의 상관 관계를 평가했습니다

4) 통계 프로그램은 SPSS for Windows 21.0 (SPSS Inc., Chicago, IL, USA)을 사용하고 p값이 0.05 미만인 경우를 통계적 유의성이 있다고 판정한다.
