# Supplementary material for: Effectiveness of oral phloroglucinol as a premedication for unsedated esophagogastroduodenoscopy: A prospective, double-blinded, placebo-controlled, randomized trial
Source: PLoS One. 2021 Aug 4;16(8):e0255016. doi: 10.1371/journal.pone.0255016 (PMC8336830; doi:10.1371/journal.pone.0255016)
Supplement: S2 File — (DOCX) [file pone.0255016.s003.docx]

**Research proposal**

**1. Research project title**

Effect of oral Phloroglucin as a premedication for unsedated esophagogastroduodenoscopy: A prospective, double-blinded, placebo-controlled, randomized trial

**2. Research institution**

**Keimyung university school of medicine, 1035, Dalgubeol-daero, Dalseo-gu, Daegu, Republic of Korea**

**3. Name and position of Research director, co-researcher**

- Research director

- Yoo Jin Lee, MD, Assistant Professor, Division of Gastroenterology and Hepatology, Department of Internal Medicine, Keimyung University School of Medicine, Republic of Korea

- Co-researcher

– Hye In Jung, MD, Clinical Assistant Professor, Division of Gastroenterology and Hepatology, Department of Internal Medicine, Keimyung University School of Medicine, Republic of Korea

- Hyun Jung Kim, MD, Clinical Assistant Professor, Division of Gastroenterology and Hepatology, Department of Internal Medicine, Keimyung University School of Medicine, Republic of Korea

- Eun Sung Choi, MD, PhD, Clinical Assistant Professor, Division of Gastroenterology and Hepatology, Department of Internal Medicine, Keimyung University School of Medicine, Republic of Korea

- Ju Yup Lee, MD, PhD, Assistant Professor, Division of Gastroenterology and Hepatology, Department of Internal Medicine, Keimyung University School of Medicine, Republic of Korea

- Kyung Sik Park, MD, PhD, Professor, Division of Gastroenterology and Hepatology, Department of Internal Medicine, Keimyung University School of Medicine, Republic of Korea

- Kwang Bum Cho, MD, PhD, Professor, Division of Gastroenterology and Hepatology, Department of Internal Medicine, Keimyung University School of Medicine, Republic of Korea

**4. Name and position of research nurse and clinical drug management pharmacist/medical device manager**

Chae Young Kim, Research nurse, Division of Gastroenterology and Hepatology, Department of Internal Medicine, Keimyung University School of Medicine, Republic of Korea

**5. Name and address of Support (request) organization, and manager information**

Not applicable

**6. Purpose**

**6.1. Priamry end point**

The proportion of participants with complete suppression of gastric peristalsis as defined by Grade 1 classification of gastric peristalsis

**6.2. Secondary end point**

1. The ease of intragastric observation which indicated the degree to which gastric peristalsis interfered with the intragastric observation

2) Comparisons of adverse events

3) The willingness of subjects to re-intake the assigned premedication

**7. Subjects**

Patients who undergo unsedated EGD

**8. Research period**

From IRB approval date to June 30, 2018

**9. Selection criteria /Exclusion criteria**

This clinical trial is conducted for patients who are all satisfied with the selection criteria and do not meet any of the exclusion criteria.

**9.1. Selection criteria**

1) A person who aged 18–80 years
2) A persion who performs unsedated EGD for the purpose of diagnosis or examination

3) A person who can understand and follow the instructions and questionnaire of clinical trials and has no communication problems

4) A person who voluntarily decide to participate in this clinical trial and give written consent

**9.2. Exclusion criteria**

1) Previous upper GI tract surgery

2) Suspected gastric outlet obstruction, deformity, or gastroparesis

3) Severe cognitive impairment

4) Hemodynamic instability

5) Suspected upper GI bleeding

6) Pregnant or lactating women

7) Upper GI mass that impaired GI motility

8) Taking medications that affected GI motility

9) American Society of Anesthesiology (ASA) physical status classification of 4 or higher

10) Declined to participate.

**9.3. Criteria for suspension and dropout**

1) If the patient withdraws consent

2) If the patient randomly stops the endoscopy or does not fill out a questionnaire

**9.4. Screening procedure**

The investigator or investigator will obtain written informed consent for this study from all patients prior to performing the procedures described in any protocol. For registration, subjects must meet selection/exclusion criteria.

**10. Sample size**

Sample size was calculated assuming a 24.6% difference in the complete inhibition rate

of gastric spasm between the placebo (11%) and trial drug (35.6%), based on a previous study

(Gastrointest Endosc 2011;73:932-41). With a significance level (α) of 0.05 and power of 80%, at least 121 participants were needed for this study. With a 10% expected dropout rate, we planned to enroll 134 participants to assure the detection of a significant difference in the primary outcome.

**11. Consent to study subjects**

Patients and guardians, if necessary, should be provided in writing after sufficient explanation and understanding of the study prior to commencement of the study.

**12. Research application method**

Patients participate in this study if they agree to the study among those who meet the selection criteria among patients who perform unsedated EGD.

**12.1. Vulnerable subject registration**

1. Reasons for including vulnerable subjects

Unsedated EGD is a safer test than sedation EGD, and recently, as there are not many patients performing unsedated EGD at tertiary hospitals, the vulnerable subjects are included in the subject's range as part of a plan for smooth test subject registration.

In addition, we want to include the elderly (over 70 years old) among vulnerable environment test subjects suggested by IRB, who are 70-80 years old because this clinical trial is available for those aged 18 or over and under 80 as of the date of obtaining written consent.

1. Countermeasures against vulnerable subjects
2. If a vulnerable subject wants to participate in this clinical trial, the investigator will fully

explain that the subject is in a vulnerable environment. Prior to obtaining consent, subjects or representatives will be given ample time and opportunity to question the details of the trial and decide to participate ..

1. It will be explained to all subjects that they can refuse to participate in the trial or give up participation at any time during the trial without loss of the benefits they can receive. In addition, test subjects will be informed that their privacy will be respected and that they will not be unfairly influenced or forced.
2. All data will be protected by removing the summary or all personally identifiable information.
3. We will not contact individuals or provide additional incentives to encourage vulnerable subjects to participate in this trial.

**13. Safety and effectiveness of the study**

There are no additional safety and efficacy issues due to this clinical trial as it is a drug currently in use in clinical practice. Since there may be nausea and vomiting caused by taking the drug, the responsible researcher will check the patient's discomfort and side effects after the examination.

**14. Test drug manager and drug administration plan**

The research is conducted by double blind using placebos of the same color and shape as produced by Daehwa Pharmaceutical Co., Ltd., which manufactures Phloroglucin(Flospan®).

The manufactured drug is managed by the clinical researcher.**.**

**15. Clinical Trial Method**

**15.1 Design of clinical trials**

1) This study is a prospective, double-blinded, placebo-controlled, randomized trial was carried out in a tertiary hospital.

2) Outpatients aged 18–80 years undergoing unsedated diagnostic endoscopy were eligible for participation in the study.

3) The participants were randomized to the test drug and placebo group and were administered the assigned premedication 15 min before EGD. Immediately after the oral administration of phloroglucinol or placebo, the standard preparation process for unsedated EGD was conducted, including the application of topical lidocaine as a pharyngeal anesthetic.

4) The endoscope was inserted 15 min after the administration of oral phloroglucinol or placebo to the participants. Two expert endoscopists performed the endoscopy procedure.

The endoscopists were blinded to participant assignments to the study groups and had no other involvement in the study. Endoscopy was performed in the morning and conscious sedation was not applied.

5) Endoscopic videos were recorded during the EGD procedures. The videos were examined by two independent video reviewers in a masked fashion to evaluate gastric peristalsis.

6) One week after EGD, all enrolled participants were asked about any adverse events encountered during the study period, including dry mouth, nausea, vomiting, dizziness, drowsiness, headache, dysuria, and voiding difficulty. Participant willingness to take the premedication again was also recorded with a “Yes” or “No” response.

**15.2. Dosage and method of drug administration for clinical trials**

160 mg of the test drug or control drug is administered orally 15 minutes before the endoscopy.

* Rationale: The adult dose of Phloroglucin solution is 160 mg and the time to reach the maximum concentration (Tmax) of oral phloroglucinol is known to be 15 min after administration.

**15.3 Assessments**

1) Evaluation of peristalsis and endoscopist's discomfort

1. Gastric peristalsis was evaluated at two time points, the first at 20 s immediately after the

insertion of the endoscope (Period A) and the last at 20 s immediately before withdrawal of the endoscope (Period B).

1. Gastric peristalsis was graded in two separate ways.

- In the first assessment, we used the “classification of gastric peristalsis,” which determined the intensity of gastric peristalsis according to the following five-point scale: Grade 1, no peristalsis; Grade 2, mild peristalsis; Grade 3, moderate peristalsis; Grade 4, vigorous peristalsis; and Grade 5, markedly vigorous peristalsis . Gastric peristalsis Grades 1 and 2 were considered to be “acceptable”.

After EGD, two board certified endoscopists (JYL and KSP) who were familiar with the above-mentioned classification of gastric peristalsis independently assessed the intensity of gastric peristalsis by reviewing the video clips.

Each time point (A and B) was graded from 1 to 5 as described with higher scores indicating more vigorous peristalsis.

- The second way of assessing gastric peristalsis was the “ease of intragastric observation,”

which indicated the degree to which gastric peristalsis interfered with the intragastric observation.

The two endoscopists who performed all of the endoscopic procedures in this trial rated peristalsis during the procedure using a score ranging from 1 to 4. The score values

were defined as 1, very easy; 2, easy; 3, slightly difficult; and 4, difficult.

1. The criteria used to evaluate gastric peristalsis in the study are presented in Table 1. There

were prepared based on existing classifications with partial modifications to enable a more

objective evaluation of gastric peristalsis

1. To ensure consistency in assessing the degree of gastric peristalsis during the study period,

22 sample video clips of routine diagnostic EGD were prepared for gastric peristalsis grading as a control exercise. One week before the start of the study, the investigators responsible for video reviews (JYL and KSP) and endoscopic procedures (HIJ and ESC) independently practiced the grading system using the sample video clips.

**Table 1. Evaluation of gastric peristalsis***

| **Classification of gastric peristalsis** |
| --- |
| ***Grade 1: No peristalsis*** |
| No or very weak gating movement of the pyloric ring is observed, but the movement does  not show a strong contraction |
| → No peristalsis |
| ***Grade 2: Mild peristalsis*** |
| A circular peristaltic wave is formed in the antrum but disappears without reaching the  pyloric ring, or circular contraction temporarily occurs immediately before the pyloric  ring |
| → Peristaltic wave does not reach the pyloric ring |
| ***Grade 3: Moderate peristalsis*** |
| A pronounced peristaltic wave is formed and reaches the pyloric ring |
| → Peristaltic wave reaches the pyloric ring, which opens and closes, showing a star-like  contraction as a result of the peristaltic wave |
| ***Grade 4: Vigorous peristalsis*** |
| Peristaltic wave is deep and pronounced and proceeds, strangulating the antrum |
| → Peristaltic wave reaches the pyloric ring, which is totally covered by the wave, and the  area exhibiting a star-like contraction protrudes towards the opening of the pyloric ring  and the mucosa is pushed out from the central part of the opening |
| ***Grade 5: Markedly vigorous peristalsis*** |
| Peristaltic wave is even deeper and more pronounced, and the entire antrum appears  severely strangled |
| → Peristaltic wave is so deep and pronounced that the antral mucosal surface is difficult  to observe because of the marked peristalsis |
| **Ease of intragastric observation** |
| ***Score 1: Very easy*** |
| → No peristalsis is noted and no interference with observation occurs |
| ***Score 2: Easy*** |
| → Mild peristalsis is noted, but observation is performed without interference |
| ***Score 3: Slightly difficult*** |
| → Peristalsis is noted and slightly interferes with observation |
| Score 4: Difficult |
| → Marked peristalsis is noted and makes observation difficult |

*This classification was adapted from the criteria of HiKi et al.

**15.4. Subject questionnaire**

Before and after the study, participants fill out a questionnaire which include patient’s character and medical history. One week after EGD, all enrolled participants were asked about any adverse events encountered during the study period, including dry mouth, nausea, vomiting, dizziness, drowsiness, headache, dysuria, and voiding difficulty. Participant willingness to take the premedication again was also recorded with a “Yes” or “No” response

The evaluation contents include the following:

1) Basic subject information-gender, age, height, weight, abdominal surgical history and comorbidity

2) Reasons for EGD, number of past EGD

3) Side effects occur after taking a test drug or a control drug

4) Participant willingness to take the premedication again

**16. Randomization assignment**

**16.1 Creation of randomized schedules**

Random assignment is a table in which the permutations of random numbers (random numbers of A and B) generated by the randomization program of the SAS system are applied sequentially from the subject number 1 (eg, group A=test group, group B=control group). Block size is not specified in this proposal.

**16.2. Randomization assignment method**

When packaging and labeling a trial drug, the manufacturer of the trial drug should properly allocate and package the test drug and reference drug according to a randomized schedule, and label the corresponding drug number (clinical drug number).

When packaging and labeling a trial drug, the manufacturer of the trial drug should properly allocate and package the test drug and control drug according to a randomized schedule, and label the corresponding drug number (clinical drug number).

Investigators are assigned randomly in the order in which they are randomized by clinical trial subjects who meet the selection/exclusion criteria, and other researchers are randomized by supplying medications labeled with the allocation number identical to those assigned by the investigator to the clinical trial subjects. This is done. Randomization allows for a 1:1 ratio between the test and control groups.

**16.3. Double-blind method**

In this clinical trial, double-blind method are covered as follows. Because the properties and method of taking the test drug and the control drug are the same, the subject and the investigator in charge of dispensing and returning the drug for clinical trial and checking compliance are blinded. In addition, independent evaluators and endoscopic doctors evaluating effectiveness are blinded. The endoscopist of this study cannot perform all tasks related to clinical trial drugs, such as dispensing and returning clinical drugs, and cannot discuss with the investigator or subject .

**17. Monitoring plan**

Data safety monitoring is conducted through a meeting between the research director and the person in charge. The storage institution for the collected data is three years after the end of the study.

**18. Research schedule**

This study is conducted on the day of the endoscopy, and the total study period is 12 months from the IRB approval date. In the first three months, the institutional review board of research ethics is reviewed and the research is conducted for the next six months. Thereafter, data collection and statistical analysis of data are conducted for one month, and a thesis is prepared in the last two months.

| Process/month | 6 | 7 | 8 | 9 | 10 | 11 | 12 | 1 | 2 | 3 | 4 | 5 |
| --- | --- | --- | --- | --- | --- | --- | --- | --- | --- | --- | --- | --- |
| 1. IRB review  2. Patient registration  3. Research progress  4. End of patient registration  5. Analysis  6.Article writing | O | O | O | O  O | O  O | O  O | O  O | O  O | O  O | O  O | O | O |

**19. Criteria for discontinuing clinical trials**

1) Early termination of the study: if the patient's enrollment is too early to reach the target number of subjects and sufficient to conduct this study

2) End of study: When the research proceeds as planned and reaches the end point

**20. Safety evaluation criteria including side effects, evaluation method and reporting method**

**20.1. Confirm clinical stability before and after the test.**

There have been no significant side effects reported for drugs to be taken by patients. No side effects and risks are expected due to the study method, but minor gastrointestinal symptoms will be checked at follow-up.

**20.2. Measures to protect the subject's safety**

Institutions that conduct clinical trials should be equipped with the necessary facilities and professional personnel for clinical trials to ensure that the trials are properly conducted as prescribed in this study protocol, and make sure that subjects are protected. The investigator should fully understand the adverse reactions and precautions specified in this plan in advance, and if a serious adverse reaction occurs during the test, stop the clinical test of the subject, take appropriate measures, and then contact the clinical research review committee.

**21. Victim compensation code**

When an unexpected accident such as an adverse reaction caused by the study occurs during the study, which treatment or hospitalization is required, the researcher bears the cost if the causal relationship is recognized.

However, for adverse reactions that occur, the subject should immediately contact the researcher so that the condition does not deteriorate.

**22. Plans for continuous safety monitoring of drugs or other procedures applied for the purpose of the study**

**22.1.** **Subject’s identification**

The initial and date of birth of all patients who selected for the clinical trial are recorded in the patient log at the time of the first visit in chronological order. If the patient is excluded from the trial, the reason should be recorded in the patient log. The interview, explanation and consent from the patients for study were obtained in an independent space. Each patient is assigned an assignment number on registration, and the assignment number and name initials are recorded on the patient case sheet.

**22.2. Recording of data and keeping**

All relevant data is entered on the case log, stored on a computer with restricted access, and stored in a lock. All data should be kept for at least 5 years after the test is over

**22.3. Data safety monitoring plan**

It is judged that there are no expected adverse reactions or specific precautions because there are no procedures or drug infusions in patients in addition to endoscopic procedures and medications that are widely used in clinical practice. Therefore, a safety inspector for monitoring will be possible as a researcher. The consent form states that it is possible to cancel at any time even after participating in the study, and the consent to participate in the study is continuously checked at each outpatient visit.

**23. Preparation of case records and subject’s privacy**

All data collected during this study should be recorded under the investigator's responsibility in case report(CRF) which was attached. In order to ensure the privacy of all subjects, this study records and evaluates by the subject identification code number assigned in the research process, and all subjects' privacy must be secured even when the results are published externally, such as publications.

**24. Statistical analysis**

1) Data were statistically analyzed using the chi-square test and independent *t*-test.

2) When the chi-square test was performed, and the expected frequency was less than five cells in 20% or more, statistical analysis was repeated using Fisher’s exact test.

3) Spearman rank correlation coefficient (r) was used to evaluate correlations between the grade of peristalsis and ease of intragastric observation at Periods A and B.

4) Statistical analyses were performed using SPSS for Windows 21.0 (SPSS Inc., Chicago, IL, USA), and differences between groups with *P*values less than 0.05 were considered to be statistically significant.
